# The clinical use of quantitative EEG in cognitive disorders

**DOI:** 10.1590/S1980-57642009DN30300004

**Published:** 2009

**Authors:** Paulo Afonso de Medeiros Kanda, Renato Anghinah, Magali Taino Smidth, Jorge Mario Silva

**Affiliations:** Reference Center of Behavioral and Cognitive Disorders of Clinicas Hospital of the University of São Paulo School of Medicine, São Paulo, SP, Brazil.

**Keywords:** Quantitative EEG, mental disorder, power spectrum, Coherence, neurodegenerative disorder, brain mapping

## Abstract

The primary diagnosis of most cognitive disorders is clinically based, but the EEG plays a role in evaluating, classifying and following some of these disorders. There is an ongoing debate over routine use of qEEG. Although many findings regarding the clinical use of quantitative EEG are awaiting validation by independent investigators while confirmatory clinical follow-up studies are also needed, qEEG can be cautiously used by a skilled neurophysiologist in cognitive dysfunctions to improve the analysis of background activity, slow/fast focal activity, subtle asymmetries, spikes and waves, as well as in longitudinal follow-ups.

## Abstract (pt)

O uso clínico do EEG Quantitativo nas doenças cognitivas. O diagnóstico das doenças cognitivas geralmente é clínico mas o EEG é importante como exame auxiliar na avaliação, diagnóstico e classificação de algumas delas. O debate atual refere-se ao uso clínico do EEGq. Embora muitos achados no EEGq ainda aguardem validação, o EEGq pode ser usado cautelosamente em situações específicas e por um neurofisiologista experiente. Nas doenças cognitivas ele pode contribuir na análise da atividade de base, em atividades focais lentas ou rápidas, assimetrias sutís, pontas e ondas e no acompanhamento longitudinal dos pacientes.

## Introduction

The primary diagnosis of most cognitive disorders is clinically based but the EEG plays a role in evaluating, classifying and following some of these disorders. The EEG is a widely accepted method for evaluating cortical information processing and neurophysiologic changes that occur during unconsciousness and varying states of conscious awareness.^1^ Moreover, it is now possible to increase EEG sensitivity through the use of Digital EEG (dEEG) and the mathematical procedures implemented in quantitative EEG (qEEG).

### Deeg differs from qEEG

DEEG is defined by the American Academy of Neurology (AAN) as the computer-based paperless acquisition and recording of EEGs, with storage in digital format on electronic media, and waveform display on an electronic monitor or other computer output device. In addition, the AAN ratifies that digital EEG is an established substitute for recording, reviewing, and storing a paper EEG record. It is a clear technical advance over previous paper methods and is highly recommended. (Class III evidence, Type C recommendation).^2,3^

### qEEG: the controversy

There is currently debate over routine use of qEEG. The AAN defines qEEG as the mathematical processing of dEEG to highlight specific waveform components, to transform EEGs into a format or domain that elucidates relevant information, or to associate numerical results with EEG data for subsequent review or comparison.^3^ Signal analysis includes: automated event detection, monitoring in the Intensive Care Unit (ICU), source analysis, frequency analysis, statistical analysis and topographic EEG displays.^4^

Unfortunately, clinical use of qEEG can be problematic particularly in the hands of untrained operators. The statistical results can be influenced by wrong electrode placement, artifact contamination, inadequate band filtering, drowsiness, comparisons using incorrect control data bases, and choice of epochs.^5^ Furthermore, statistical processing can yield a large numbers of statistical abnormalities, not all of which are of clinical relevance. These are some reasons, despite the volume of published data, that the clinical usefulness of qEEG remains controversial.

Nevertheless, despite the fact that many findings concerning the clinical use of qEEG are awaiting validation by independent investigators, and that confirmatory clinical follow-up studies are needed, qEEG can be cautiously used by a skilled neurophysiologist in cognitive dysfunctions to improve the analysis of background activity, slow/fast focal activity, subtle asymmetries, spikes and waves, as well as in longitudinal follow-ups. Although some cooperation from most patients is needed, EEG has high test-retest reliability and reflects physiological cortical function where these properties render qEEG frequency change measurements both a practical and useful adjunct to neuropsychological tests. The question remains as to whether qEEG constitutes an investigational method or can be considered an established addition to dEEG in routine clinical use.

This aim of this paper was not to provide a comprehensive review of qEEG literature but to briefly discuss selected topics on the practical clinical use of qEEG in disturbances of consciousness. The topics of evoked potentials and epilepsies are beyond the scope of this paper.

The qEEG can explore physiological and pathological correlates of conditions where consciousness is normal^6^ or impaired, quantifying the increase in low-frequency components of the background activity^7,8^ using coherence analysis to study the neural network functional state.^9^ The method can compare groups of diseases using power spectra^10^ and apply three-dimensional source localization methods to identify the generators of pathological EEG activity.^11^ Thus, there is a broad range of applications where qEEG can be used as a tool to improve clinical diagnosis, evaluation and conduct: encephalopathies; delirium; learning disabilities; attention disorders; mood disorders; ICU monitoring and dementia.

### Encephalopathies and delirium

Quantitative EEG is described as a tool for evaluating encephalopathies associated to diverse causes including Creutzfeldt-Jakob disease,^12,13^ uremic,^14^ hypoxic-ischaemic,^15^ hepatic,^16-18^ methamphetamine abstinence,^19^ baclofen overdose,^20^ acute lymphoblastic leukemia^21^ and coma.^22^ The method was also used to describe encephalopathy associated to poisoning in the Chernobyl accident.^23^

The encephalopathies are frequently accompanied by delirium which is, in most cases, symptomatic of a serious underlying disease. Thus, the diagnosis of the delirium is critical and urgent because of life-threatening medical complications associated with its high morbidity and mortality. As delirium often goes undiagnosed or misdiagnosed, qEEG has considerable potential in several specific groups, not only for confirming the clinical diagnosis of an organic syndrome, but for distinguishing delirium from dementia. Relative power in the alpha frequency band enables qEEG to distinguish normal from encephalopathic subjects. The variables best able to distinguish delirious from non-delirious patients include the amount of EEG theta activity, relative power in the delta frequency band, and the amount of activity in the slow wave bands compared to the alpha band.^24-26^

In sum, there is a considerable reference list on the subject. However, the views of the American Academy of Neurology (AAN) and the Brazilian Neurophysiology Society (SBNC) on the issue should be considered. According to the AAN and SBNC and based on Class II and III evidence, the frequency analysis, in expert hands, may be a useful complement to the EEG in encephalopathies in cases where the diagnosis remains unresolved.^3,4^

### Learning disorders

Many studies have shown the value of qEEG in complementing the investigation of learning disabilities^27,28^ and evaluating learning disorders, where qEEG discriminant accuracy ranged from 46% to 98%.^29^ The models studying the correlations between intelligence and EEG measures can be tested by two categories of EEG parameters: 1. EEG power and; 2. EEG network properties such as coherence and phase delays and non-linear dynamical models of network complexity.

#### EEG power

In a neurophysiological sense, EEG power represents the sum of neurons discharging synchronously. The thickness of the cortical layer is positively correlated with intelligence so it is possible that EEG power may also be a measure that reflects the capacity or performance of cortical information processing. This occurs in a complex and partly non-linear fashion, and is influenced by a variety of factors such as the thickness of the skull, cerebrospinal fluid, inter-electrode distance and age.^30^ Despite these facts, some EEG power studies using LORETA and surface EEG reported a positive correlation between IQ and increased absolute alpha and beta band power,^31^ and decreased delta and theta band power.^32^ Further, some EEG network studies have argued that increased complexity and neural efficiency are positively related to intelligence.^33-35^ Thus, there is a negative correlation between EEG coherence and IQ especially in the frontal lobes.^36,37^

Therefore, a continuum of relationships between EEG and cognitive function have been reported in some studies, which have shown significant correlation between EEG and intelligence thus demonstrating predictive validity between EEG and neuropsychological performance.^33,38,39^ In general, the higher the absolute amplitude or power of the EEG then the higher the IQ^37,40^. Also, the higher the severity of the learning disability, the greater the qEEG clinically-significant abnormalities^41,42^ where high value of slow power is associated with low IQ.^43^

#### Relationship between EEG coherence and intelligence

Coherence is an amplitude-independent measure that analyses the phase consistency between two time series and the network properties of a system. Low coherence is positively correlated with IQ and is a predictor of IQ.^43,44^ This indicates that the more complex the neural network, the higher the spatial differentiation, with lower coherence between different neuron pathways.^45^

#### EEG phase ‘delay’ and intelligence

Phase angle is the lag delay between two time series (in this case sets of electrodes) and varies as a function of electrode distance and independently of the amplitude of the two time series, where the ability to synchronize distributed generators is significant while the number of connections or strength of connections may have less relevance. The limit of the shortest phase is equal to 0 or near-zero and the studies of frontal lobe phase delay have shown that the shorter the phase delay, the higher the IQ.^29,46,47^ The position of the American Neuropsychiatric Association (ANA)^29^ is that qEEG is capable of providing accurate probability estimates of the likelihood that a given patient has attentional or learning disabilities, on the basis of several replicated studies, if the individual patient assessed matches the selection criteria of the patient group used to form the discriminant. In contrast, the AAN and SBNC deem the qEEG an investigational tool for clinical use in learning disability (Class II and III evidence, Type D recommendation).^3,4^

### Attentional disorders

Attention-deficit hyperactivity disorder (ADHD) is a common neuropsychological disorder of childhood affecting 3–5% of school-aged children.^29^ Consequently, there is great interest in developing an accurate neurophysiologic diagnostic test differentiating ADHD from normality and other pediatric mental disorders. A literature search on MEDLINE (1997 through 2008) examining EEG associated with the terms “attention deficit” or “hyperactivity” or” hyperkinetic” retrieved 1214 articles, and there are promising results. For instance, children and adults diagnosed with ADHD have high slow-wave power (delta and theta) and children and adolescents with ADHD seem to have reduced beta power compared with their respective normal controls.^48-50^

Meta-analytic results support an ADHD trait of Cz electrode (eyes-open, fixed-gaze) theta/beta ratio increase in comparison to controls yielding 86–90% sensitivity and 94–98% specificity.^51^ Despite this, the generalization of the results is limited because theta/beta changes and increased theta can be found in other neurologic and psychiatric conditions. Thus, the emphasis may be on the integration of the EEG as supplemental information in the complete clinical picture.^52^ This means that among ADHD patients there is an increased theta/beta ratio but not all patients with this trait are candidates for ADHD. Given these limitations, controversy remains where the ANA favors the clinical use of qEEG in ADHD^29^ while the AAN and the SBNC are against its routine clinical use (Class II and III evidence, Type D recommendation).^3,4^

### Mood disorders

#### Depression

Conventional EEG (or dEEG), per se, shows from 20% to 40% abnormalities in depressed patients. Although unspecific, these changes help in differentiating a normal or nearly normal EEG of depression from a similarly impaired patient with severe EEG slowing suggestive of functional or structural decline regardless of diagnosis. Therefore, an abnormal EEG can identify patients at greater risk for functional decline. Consequently, it can be a useful tool for evaluating depression.^53^ Furthermore, there are a considerable number of publications investigating qEEG in depression, and some studies were replicated across academic institutions^54^. Among the trait markers is frontal alpha asymmetry,^55,56^ changes in frontal qEEG cordance,^57,58^ asymmetry in frontotemporal slow-wave activity,^59^ decreased inter-hemispheric coherence in the delta and/or theta frequency bands,^60,61^ increased delta and theta bipolar absolute powers of the right hemisphere;^62^ higher percentage of theta in posterior brain areas^63^ and changes in beta activity.^64-66^ The accuracy of these qEEG findings in detecting depression has been demonstrated and replicated in large samples with 72–93% sensitivity and 75–88% specificity.^29^ Some caution must be exercised, however, in generalizing results, because of the great number of possible psychiatric diagnostic subcategories into which a patient might be placed, and due to the use of antidepressant medication and lack of a standardized methodology. The ANA recommends the use of qEEG as an additional tool classifying unipolar and bipolar patients, differentiating between healthy and depressed individuals, and for distinguishing depression from cases of dementia, schizophrenia, and alcoholism.^29^ The AAN and SBNC, on the contrary, have a more reserved view and state that in depression, qEEG remains investigational (Class II and III evidence, Type D recommendation).^3,4^

#### Panic disorder, schizophrenia, obsessive-compulsive disorder and anxiety

At present the clinical usefulness of quantitative spectral analysis of the EEG (QEEG) in the diagnosis of these psychiatric conditions remains controversial, based on the lack of “Class I evidence or overwhelming Class II evidence”.^3,4^

### Intensive care unit (icu) and operating room (or) monitoring

Spectral analysis may supplement dEEG in situations when a graphic display can identify and clinically measure changes more reliably.^3^ Carotid endarterectomy, continuous monitoring for early detection of acute intracranial complication during cerebrovascular surgery and other situations where the cerebral blood flow is compromised, are examples. qEEG has shown its value in the early diagnosis and management of severe acute cerebral infarctions and post-SAH vasospasms. In comatose patients, it can provide diagnostic and prognostic information which is otherwise unobtainable.^67^ qEEG seems to be useful for helping clinicians to decide the optimal time-point to disconnect the patient from the ventilator.^68^ The mathematical tools of the qEEG reduce evaluation time of the recorded exam whereas spectral analysis allows bedside non-expert staff to recognize EEG changes in a timely fashion.^69,70^ Therefore, the AAN and SBNC suggest the use of qEEG in ICU patients at high risk for ischemic stroke, acute intracranial bleed, vasospasm, critically elevated intracranial pressure (ICP), or related ischemia, detection and management of convulsive and non-convulsive status epilepticus in high-risk patients, titration of barbiturates, anti-epileptics given for non-convulsive status and mannitol given for increased ICP. On the basis of considerable Class II evidence, EEG seizure detection and frequency analysis is considered an established option when used as an adjunct to routine or digital EEG for continuous brain monitoring by frequency trending in the OR or ICU to detect early acute intracranial complications, and for screening for possible epileptic seizures in high-risk ICU patients (Type B recommendation).^3,4^

### Dementia

Visual analysis of EEG is a helpful auxiliary method in Alzheimer’s disease (AD) diagnosis.^4^ The most frequent EEG findings are the displacement of background frequency into delta and theta ranges and the decrease or dropout of alpha central frequency.^71^ However, these EEG changes usually occur in moderate and advanced stages of the disease. Accordingly,^72^ an inverse correlation between the degree of cognitive impairment and the power of low frequency electrical activity in the EEG was observed. Since the first studies of qEEG,^73,74^ the spectral analysis and other statistics have been applied to EEG. A decrease in alpha and beta activities have been observed in various studies published over the last decade.^75-77^ Furthermore, the “alpha like” rhythm could be a diagnostic marker,^77^ since there is a decrease of the alpha frequency to 6.0–8.0 Hz in mild AD patients. Another high sensitivity aspect in qEEG is the background spectral analysis that agrees strongly with the clinical diagnosis of AD. The sensitivity of the spectral analysis ranges from 71% to 81% in several studies^75,78-80^ and the spectral analysis also presents strong correlations with neuropsychological tests.^80^ Another qEEG tool is called Coherence (Coh) Analysis, which evaluates the level of covariance between spectral measures obtained by any given pair of electrodes. High Coh has been considered evidence of structural and functional connections between brain cortical areas.^81^ Coh studies aid understanding of the functional relationships among brain areas, which may vary under different conditions. Among alternative techniques for studying relationships between brain areas, Coh is a well established method, used in the quantification of hemispheric connectivity “through the corpus callosum”, both in awake and sleeping patients.^82,83^ Leuchter and colleagues^84^ studied AD and VaD patients comparing them to control subjects, and found decreases in Coh in both AD and VaD. Besthorn et al.^85^ studied 50 patients with AD and found a decrease in Coh in theta, alpha and beta bands versus control subjects, in central and frontal areas. Their findings were comparable to results of Locatelli et al.^86^ who showed an alpha band Coh decrease in AD in left temporo-parieto-occipital areas. The Brazilian Medical Association (AMB) and Brazilian Clinical Neurophysiology Society (SBNC) 2008 guideline refers to the conventional EEG as an established instrument in the evaluation of dementias (Type B recommendation). In addition, frequency analysis (qEEG) is a useful tool to improve the detection of slow waves (Type B recommendation). It can show an increase of theta waves and decrease of alpha and beta waves in AD patients compared with normal subjects (Type B recommendation). Frequency analysis also has predictive value concerning the development of cognitive impairment in dependently from clinical parameters (Type C recommendation). Moreover, there is a strong correlation between some qEEG dipole sources characteristics (from the usual EEG bands) and cognitive functions quantified on some specific AD evaluation scales (Type B recommendation). The combined use of such qEEG parameters and cognitive scales is recommended to improve the detection of dementia (Type B recommendation). qEEG can be used as a tool in dementias in much the same way as Single photon emission computed tomography (SPECT) (Type B recommendation) and MRI (Type A recommendation). These methods are not mutually exclusive, but rather, are complementary.^87^

#### qEEG pitfalls and caveats

qEEG abnormal patterns can be regarded as a specific sign of brain dysfunction. Further, delta and theta slowing are frequently associated with cortical atrophy. However, the same qEEG abnormalities can be found in many different disorders. Quasi pathognomonic patterns of any specific disorder are a rare occurrence. Nevertheless, EEG is an integral part of the diagnostic process in many diseases. Consequently, several qEEG systems have been approved by the U.S. Food and Drug Administration (FDA) for the post-hoc analysis of EEGs and are classified as Class II devices.^88^ In addition, a literature search on the MEDLINE database (1997 through 2008) using the terms quantitative EEG or qEEG retrieved 1545 articles. So, in spite of this volume of published data, why is the method not widely used, at least among neurologists? At present, there are legitimate scientific debates and differences of opinion concerning some uses of qEEG for a multitude of reasons.

Firstly, because there is a lack of a paradigm or unified methodology to handle the huge amounts of data generated when the EEG is recorded. Each researcher has their own mathematical tools hampering comparison of results among laboratories. This incompatibility prevents the formation of a new, coherent and interchangeable knowledge data base as has occurred in dEEG. Secondly, one of the major problems in electroencephalography is the intra/inter-subject variability. Unfortunately, the EEG is subject to great variability depending on biological (age, vigilance, thickness of tissues), technical (AC or DC current equipment, electrodes, gel characteristics, impedances) and artifactual issues. Ideally, a feature should be stable and recur in the same and other patients in order to be of clinical use. Therefore, in the qEEG field each new postulate requires large dataset to allow a full investigation to be carried out. Thirdly, it is not difficult to group individuals with the same diagnoses and to then find electroencephalographic similarities among them. More challenging however, is to select an individual randomly and match them with a given group. Thus, the aim of this technology could be, instead of providing diagnoses, to complement the findings of a given diagnosis and to aid follow-up in specific cases. Last but not least, electroencephalography is a very specific field in which years are required to become a specialist. Thus, it is often difficult for professionals other than neurophysiologists to make sense of all the charts and tables generated by the up-to-the-minute software available.

In sum, clinical diagnosis of cognitive dysfunction is a complex process depending on multiple sources of information. Taking this into account, computer-assisted diagnosis using qEEG is an accurate, inexpensive, easy to handle tool that represents a valuable aid for diagnosing, evaluating, following-up and predicting response to therapy.

## Appendix

### Strength of recommendation ratings

**Type A.** Strong positive recommendation, based on Class I evidence, or overwhelming Class II evidence.

**Type B.** Positive recommendation, based on Class II evidence.

**Type C.** Positive recommendation, based on strong consensus of Class III evidence.

**Type D.** Negative recommendation, based on inconclusive or conflicting Class II evidence.

**Type E.** Negative recommendation, based on evidence of ineffectiveness or lack of efficacy.

### Standards

Generally accepted principles for patient management that reflect a high degree of clinical certainty (i.e., based on Class I evidence or, when circumstances preclude randomized clinical trials, overwhelming evidence from Class II studies that directly address the question at hand, or from decision-analysis that directly addresses all the issues).

### Guidelines

Recommendations for patient management that may identify a particular strategy or range of management strategies that reflect moderate clinical certainty (i.e., based on Class II evidence that directly addresses the issue, decision analysis that directly addresses the issue, or strong consensus of Class III evidence).

### Practice options or advisories

Other strategies for patient management for which there is some favorable evidence, but for which the community still considers this an option to be decided upon by individual practitioners.

### Practice parameters

Results, in the form of one or more specific recommendations, from a scientifically-based analysis of a specific clinical problem.

### Quality of evidence ratings

**Class I.** Evidence provided by one or more well-designed, prospective, blinded, controlled clinical studies.

**Class II.** Evidence provided by one or more well-designed clinical study such as case control, cohort studies, etc.

**Class III.** Evidence provided by expert opinion, non-randomized historical controls or case reports of one or more patients^3^.
